# Genomic Alterations Affecting Competitive Endogenous RNAs (ceRNAs) and Regulatory Networks (ceRNETs) with Clinical Implications in Triple-Negative Breast Cancer (TNBC)

**DOI:** 10.3390/ijms25052624

**Published:** 2024-02-23

**Authors:** Amal Qattan

**Affiliations:** 1 Department of Molecular Oncology, King Faisal Specialist Hospital and Research Centre, Riyadh 11211, Saudi Arabia; akattan@kfshrc.edu.sa; 2 College of Medicine, Alfaisal University, Riyadh 11533, Saudi Arabia

**Keywords:** triple-negative breast cancer (TNBC), ceRNAs, ceRNETs, ceRNome, microRNAs, circRNAs, lncRNAs, biomarkers, therapeutic targets

## Abstract

The concept of competitive endogenous RNA regulation has brought on a change in the way we think about transcriptional regulation by miRNA–mRNA interactions. Rather than the relatively simple idea of miRNAs negatively regulating mRNA transcripts, mRNAs and other non-coding RNAs can regulate miRNAs and, therefore, broad networks of gene products through competitive interactions. While this concept is not new, its significant roles in and implications on cancer have just recently come to light. The field is now ripe for the extrapolation of technologies with a substantial clinical impact on cancer. With the majority of the genome consisting of non-coding regions encoding regulatory RNAs, genomic alterations in cancer have considerable effects on these networks that have been previously unappreciated. Triple-negative breast cancer (TNBC) is characterized by high mutational burden, genomic instability and heterogeneity, making this aggressive breast cancer subtype particularly relevant to these changes. In the past few years, much has been learned about the roles of competitive endogenous RNA network regulation in tumorigenesis, disease progression and drug response in triple-negative breast cancer. In this review, we present a comprehensive view of the new knowledge and future perspectives on competitive endogenous RNA networks affected by genomic alterations in triple-negative breast cancer. An overview of the competitive endogenous RNA (ceRNA) hypothesis and its bearing on cellular function and disease is provided, followed by a thorough review of the literature surrounding key competitive endogenous RNAs in triple-negative breast cancer, the genomic alterations affecting them, key disease-relevant molecular and functional pathways regulated by them and the clinical implications and significance of their dysregulation. New knowledge of the roles of these regulatory mechanisms and the current acceleration of research in the field promises to generate insights into the diagnosis, classification and treatment of triple-negative breast cancer through the elucidation of new molecular mechanisms, therapeutic targets and biomarkers.

## 1. Introduction

MicroRNAs (miRNAs) are known to negatively regulate gene product expression through binding to miRNA response/regulatory elements (MREs) in the 3′ UTR followed by the transcript degradation or inhibition of translation. We now know that the interaction of miRNAs and mRNAs is bidirectional, driven by competition for miRNA binding by RNA species containing the same MRE. The idea of competitive endogenous RNAs (ceRNAs) has existed for over a decade, but our understanding of it as a critical and consequential institution of complex transcriptional regulation is just now coming of age, particularly as it relates to disease. Over the past decade, the association of an aberrant expression of ncRNAs with tumorigenesis and malignant progression, with implicated RNAs having effects on genomic stability, proliferation, survival, migration and cellular homeostasis, has been noted [1]. Indeed, in triple-negative breast cancer (TNBC), circular RNAs (circRNAs) and long non-coding RNAs (lncRNAs) have been implicated as decoys for regulatory factors, such as miRNAs and transcription factors, that affect gene expression and, consequently, disease initiation and progression [2,3,4]. In addition, circRNAs and lncRNAs have been found to affect chemotherapy and radiotherapy resistance in TNBC and have been proposed as therapeutic targets [2,3]. Pseudogenes can encode a class of ceRNAs and are often found in non-coding regions, which are commonly altered in the copy number in breast cancer [5]. In a study to identify the gene–pseudogene pairs involved in breast cancer pathology, functionally relevant candidate genes and their interacting miRNAs were used to delineate ceRNA networks in breast cancer cell lines and patient-derived tissues [6]. The pseudogene GBP1P1 (guanylate binding protein 1 pseudogene 1) was found to enhance GBP1 gene expression through the modulation of miR-30d-5p, thereby contributing to the viability, migration and clonogenicity of breast cancer cells. While this study was not specific to TNBC, it illustrates the involvement of pseudogene ceRNA networks in breast cancer pathology. These discoveries have introduced an even more complex system of post-transcriptional regulation that is likely to affect the pathogenesis of TNBC and other cancers and ceRNA networks. These networks may amplify the impact and importance of genomic alterations affecting network components, including signaling factors, transcription factors, transcripts, decoy RNAs and miRNAs. While the study of the role of ceRNA networks in TNBC is relatively young, there is a burgeoning and compelling rationale for the investigation of their impact on the disease and their potential utility as biomarkers.

### 1.1. Decoding the Competing Endogenous RNA (ceRNA) Hypothesis

**Figure 1:**
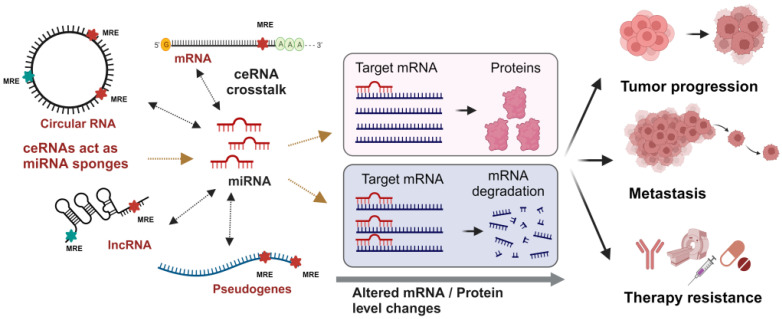
ceRNA interaction networks in gene regulations: MicroRNAs play a crucial role in gene regulation by negatively influencing the expression of mRNAs. Through targeted and specific interactions, miRNAs modulate gene expression by binding its seed region (nts 2–8) to the 3′ untranslated region (3′UTR) of the target mRNA, called miRNA response/regulatory elements (MREs). Conversely, a reverse relationship where mRNAs and other endogenous RNAs, like circRNAs, lncRNAs and pseudogenes possessing the same MRE sequence, can act as miRNA sponges and could competitively bind and sequester miRNAs, thereby limiting their availability for other target mRNAs. Interaction between a miRNA and multiple target RNAs leads to competitive endogenous (ceRNA) crosstalk, further contributing to the complexity of post-transcriptional gene regulation. This consequential impact on protein availability has the potential to drive tumor progression, facilitate metastasis and may foster resistance to therapeutic interventions.

Salmena et al. first introduced the concept of ceRNA in 2011 [7]. This hypothesis states that the effectiveness of miRNAs in regulating transcript levels depends on the concentration of competing MREs. MREs reside within the 3′ UTR of the transcript, which can vary in length and be expressed with the coding sequence or independently, affecting the balance of competition for miRNA binding [8]. Competing RNA species include mRNA, circRNA, lncRNA, tRNA, pseudogene RNA and ribosomal RNA (rRNA); see Figure 1. Broader categories within the “ceRNome” include the products of genes that are protein coding, pseudogenes, and those encoding long non-coding RNAs [9]. Competitive regulatory networks are formed with the involvement of various ncRNA types, which compete with each other, including circRNA-miRNA-mRNA ceRNETs, pseudogenes-miRNA-mRNA ceRNETs, and lncRNA-miRNA-mRNA ceRNETs. Conversely to the paradigm of miRNAs negatively influencing the expression of mRNA, a reverse relationship also exists where mRNAs and other ncRNA species influence miRNA activity. Those sharing multiple MREs compete for the binding of miRNAs, causing reduced inhibitory activity of the miRNAs as the concentration of MREs increases [7,10]. This can be mediated by “artificial transcripts”, non-mRNA species containing repeated MREs that act as “miRNA sponges’’, resulting in a complex system of post-transcriptional regulation mediated by interactions between RNA species [7,10]. Another perspective on ceRNA crosstalk is that it is based on interactions between the miRNA and two target RNAs (mRNA or ceRNA). The steady state of this dynamic is a result of the rates of transcription and degradation of the miRNA and targets and the rates of association/dissociation and degradation of the miRNA/target complex [11]. A change in the transcription of a miRNA or its target RNAs can alter the entire regulatory network. Thus, ceRNA networks and transcription factors are interrelated, and each is responsive to the altered expression and activity of the other [11]. As such, alteration of one component of the network may cause an amplification of effect through secondary modification of the broader regulatory network. This phenomenon indicates that pathogenic genomic alterations or specific alterations in the expression or activity of a transcription factor or signaling factor, such as in cancer, may have a broader and more robust effect than anticipated since ceRNA and post-transcriptional regulatory networks may be affected.

### 1.2. The Importance of Studying Genomic Alterations Affecting ceRNAs and ceRNETs in TNBC

Triple-negative breast cancer (TNBC) is an aggressive breast cancer subtype that does not express the estrogen receptor (ER), epidermal growth factor receptor 2 (HER2) or progesterone receptor (PR). This subtype is also relatively challenging to treat (being an untargetable disease) because of the absence of these molecular targets and broad heterogeneity in molecular and regulatory pathway profiles [12]. Recent evidence suggests that molecular differences among TNBC tumors affect and are affected by ceRNA networks. In TNBC, the dysregulation of ceRNAs has been implicated in the increased expression of pro-tumorigenic factors, as is discussed further below, although the roles of ceRNAs in TNBC pathogenesis and progression remain to be fully understood. The implications of this potentially powerful regulatory mechanism regarding a response to therapy is another compelling current area of research, although research into ceRNA regulation networks, including those involved in TNBC and other cancers, is young and evolving.

DNA-damage-response (DDR) defects in TNBC lead to characteristic genomic instability and alteration of both coding and non-coding DNA [13]. Genomic alterations, such as copy number variation (CNV), mutation, translocation and fusion, can affect ceRNA networks. The consequences of an altered transcription of coding genes become more complex when considering that the resulting mRNAs may be competing in ceRNA networks regulating the expression of other relevant genes. Further, alteration in non-coding genes is an important but previously overlooked mechanism of post-transcriptional regulation in cancer. Specific CNV-altered lncRNAs with corresponding expression alterations have been identified in breast cancer [14]. Some of these lncRNAs regulate functions that are key to cancer pathogenesis and therapy, such as growth signaling, immune infiltration and immune checkpoints [14]. In addition to transcriptional regulating functions, including binding to RNA-binding proteins and RNA polymerase II, circRNAs that may be affected by genomic alteration can influence ceRNA networks by acting as miRNA sponges [4]. Specific dysregulated ceRNAs may also be prognostic or diagnostic biomarkers. For example, several circRNAs have been implicated as potential markers in TNBC [3]. Perhaps more importantly, new targets for therapy are likely to be discovered in the study of ceRNAs in TNBC. Targeting specific ceRNA sequences or, as some evidence suggests, the function-conferring secondary structure of ceRNAs [15], may be a potent modality of therapy. Herein, we discuss the clinical implications of our burgeoning knowledge of ceRNA networks in TNBC.

TNBC includes multiple molecular and phenotypic subtypes, each with its own prognostic and therapeutic profile. The classification system described by Lehmann et al. in 2016 includes immunomodulatory (IM), mesenchymal (M), mesenchymal stem-like (MSL), basal-like 1 (BL1), basal-like 2 (BL2) and luminal androgen receptor (LAR) [16]. BL1 exhibits an increased expression of cell-cycle and DNA-damage-response genes (DDR), and BL2 exhibits increased myoepithelial markers and growth factor signaling [17,18]. Since androgen receptor (AR) expression is less prevalent in TNBC than other breast cancer subtypes and AR regulates multiple molecular pathways affecting tumorigenesis and disease progression, the molecular classification of TNBC according to AR expression has gained interest. Quadruple-negative breast cancers (QNBC: TNBC lacking AR expression) have molecular and phenotypic features that are different from AR-positive TNBC tumors [12]. These differences between molecular subtypes equate to variations in pharmacologic approaches to the management of TNBC. Given this heterogeneity among TNBC cases, it is likely that there is heterogeneity in ceRNAs and ceRNETs in TNBC, with potential clinical implications. There is a compelling argument that targeting key elements in ceRNA networks to mitigate altered molecular profiles and signaling programs would be more effective than attempting to single out and target downstream molecular factors that characterize each subtype. As an inroad to understanding the differences in ceRNA networks among breast cancer subtypes, investigators may begin with existing knowledge of dysregulated miRNAs. To that end, differential dysregulation of miRNAs targeting PI3K, Myc, TP53, SOD2 and cell-cycle checkpoint factors has been identified among breast cancer subtypes [12].

This review focuses on the genomic alterations influencing competitive endogenous RNA networks (ceRNAs) and their regulatory networks (ceRNETs) in TNBC, with a particular emphasis on understanding the mechanisms, as well as compelling research questions and clinical implications. With the discussion of these regulatory networks, we hope to inform the development of valuable biomarkers and therapeutic targets for TNBC.

## 2. Genomic Alterations Affecting ceRNAs and ceRNETs in TNBC

Significant mutations and copy number variations are associated with TNBC, with a large amount of heterogeneity within the subtype [19]. Compared to HER2+ and HR+ (ER+ or PR+) breast cancer, TNBC has been found to harbor more numerous and differing genomic alterations, which have more pronounced implications on mutational burden, immune activation and prognosis [19]. Such alterations can be predicted to affect the expression of transcripts and UTRs, leading to differences in the abundance of MREs. Such changes are likely to affect the balance of competition for miRNA binding and, therefore, substantially affect the ceRNA network regulation of transcripts, contributing to tumorigenesis, tumor progression and drug response. Examples of such alterations affecting the balance of ceRNA networks and, ultimately, disease phenotypes and outcomes, have been increasingly reported in the literature. Thus, it is a timely endeavor to understand the ceRNA networks involved in TNBC’s development and progression, the mechanisms of their alteration and how they can be exploited for better management of TNBC.

### 2.1. Types of Genomic Alterations and Their Effect on ceRNAs and ceRNETs

**Figure 2:**
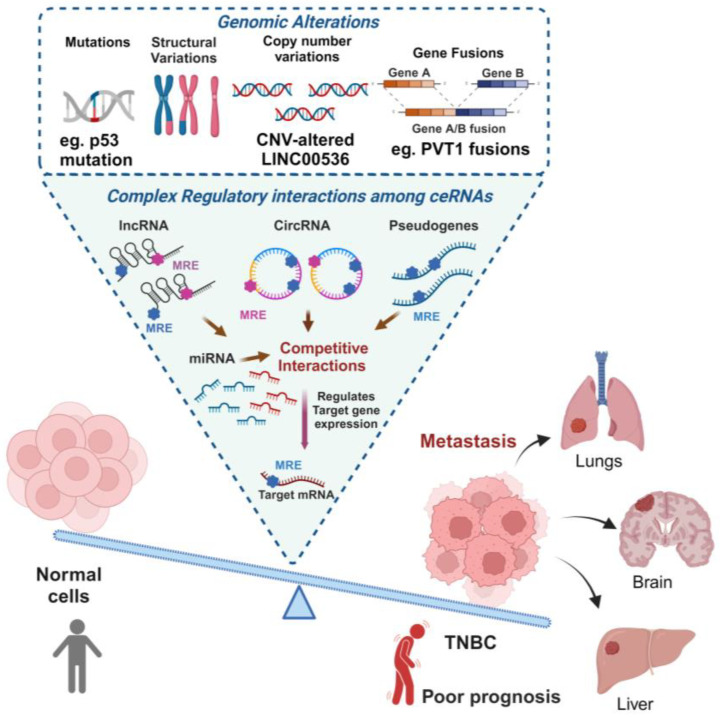
Overview of aberrant ceRNA network regulations caused by genomic alteration in TNBC. Aberrant genomic alterations, manifested in various forms like mutations, structural variations, copy number variation (CNV), gene fusions and more, can play a crucial role in driving TNBC, leading from uncontrolled cell growth and proliferation to metastasis. These dysregulations impact the complex regulatory interactions among competitive endogenous RNA (ceRNA) networks. CeRNAs, including various RNA molecules, like circular RNAs (circRNAs), long non-coding RNAs (lncRNAs), pseudoRNAs and messenger RNAs (mRNAs), compete for the shared microRNAs using microRNA response/regulatory elements (MREs), thereby influencing each other’s expression levels, and also influence the expression of specific downstream genes involved in cancer-related pathways. Aggressive behaviour, metastatic potential and a poor clinical outcome associated with TNBC highlight the significance of understanding genomic alterations and their downstream complex regulatory interactions among ceRNA networks.

Dysregulation of RNA expression is a central mechanism of ceRNA regulatory network alteration. The altered expression of lncRNAs, some of which have been implicated as competitive for miRNA binding, has been observed in TNBC [20], as discussed in more detail below. Likewise, altered miRNA expression, which commonly occurs in many cancers, including TNBC [21], can disrupt ceRNA networks. Specific examples of this are also discussed below. There are several mechanisms by which genomic alteration can affect ceRNA expression and the balance of ceRNA networks. An overview of these changes that may affect TNBC is presented in Figure 2.

One mechanism by which alterations within the genome can affect ceRNA networks is through the mutation of genes that affect the expression of miRNAs and ceRNAs. In TNBC, TP53 is often mutated [22]. This transcription factor regulates the expression of several miRNAs and lncRNAs depending on its status as deleted or mutated [23]. These RNAs constitute a network that regulates the epithelial–mesenchymal transition (EMT) with p53, promoting an epithelial gene expression program in breast cancer, e.g., through the targeting of ZEB1/2 EMT transcription factors [23]. These transcription factors also mediate negative feedback on p53 through the ceRNA network. Another network of miRNAs and lncRNAs mediate p53 regulation of the EMT transcription factors SNAIL, SLUG and TWIST [23]. The miR-200 family, which is regulated by p53, is central to these networks. Multiple studies have implicated a high miR-200 expression as being associated with poor outcomes in multiple cancer types, including breast cancer [24]. Also, in hepatocellular carcinoma cells, a ceRNA network centered on five miRNAs was found to be integral to p53-mediated phenotypes [25].

The HULC/miR-200a-3p axis and other ceRNA regulatory mechanisms affecting p53 function and DNA repair have been revealed in TNBC [26]. In this work, multiple ceRNA networks were identified by the microarray analysis of mRNAs and lncRNAs from normal and TNBC tissues using weighted gene co-expression analysis. One of the identified networks, which were functionally enriched for p53, proliferation and DNA repair, highly correlated with the Ki-67 status. Two of the co-regulated mRNAs (RAD51AP1 and TYMS) in the network were found to correlate with overall survival. The network is complex, involving around 50 lncRNAs competing with eight miRNAs. Finding relevant targets in the network may be best accomplished by focusing on the miRNAs directly regulating RAD51AP1 and TYMS (Thymidylate Synthase); for example, hsa-miR-3163, regulating TYMS and its competitor lncRNA PCA3. Both PCA3 (previously known as DD3) and TYMS have been shown to be overexpressed in prostate cancer [27,28], and TYMS was reported as correlating with aggressiveness [28]. Overexpression of TYMS was associated with deletions at 5q21 (*p* < 0.0001), 6q15 (*p* < 0.0001) and 3p13 (*p* = 0.0083) [28].

The mutation of any of a multitude of transcription factors or regulators of miRNA can be imagined to potentially affect the balance of the ceRNA networks since they affect the abundance of specific mRNAs, miRNAs, circRNAs and lncRNAs. BRCA mutations are present in a subpopulation of breast cancer patients. BRCA1, mutated in TNBC cases, can epigenetically suppress the activity of miRNAs, for example, miR-155, which is positively associated with breast cancer tumorigenesis and metastasis [29,30,31]. To complicate the repertoire of mechanisms further, transcription factors can alter the polyadenylation of transcripts using alternate sites, ultimately changing the length of the 3′UTR [32]. Intuitively, shorter 3′UTRs potentially carry fewer MREs, making them less competitive for miRNA binding. A shortening of 3′UTRs that constitute ceRNAs has been shown to affect ceRNET crosstalk and the expression of specific genes [8]. The most frequently mutated genes in TNBC include TP53 (78.20%), PIK3CA (11.70%), PTEN (6.70%) and BRCA1 (5%), according to cBioportal data (Table 1) [33]. While BRCA1/2 mutations are less common, they have higher penetrance. Interestingly, specific ceRNA network alterations have been identified in patients with BRCA mutations, as recently, the circHIPK3 (circular RNA homeodomain-interacting protein kinase 3)-based ceRNA network was discovered in this population, which contributed to poor prognosis [34].

### 2.2. Copy Number Variation (CNV) Altered ceRNAs and ceRNETs

CNV is a substantial factor in the progression of cancers, including breast cancer [14], and has been associated with lymph node metastasis in TNBC [35]. While CNV of lncRNAs is less common than that of coding genes, CNV in intergenic regions is quite common, accounting for over 60% of CNV in breast cancer [5]. These intergenic regions are residential for non-coding ceRNAs, such as pseudogenes. An example of ceRNAs affected by CNV is the circRNA circBIRC6, which promotes pluripotency [36]. Specific examples of CNV-altered ceRNA species have been identified in breast cancer and TNBC. CNV-altered ceRNET-associated mRNAs in breast cancer were shown to have a biological function and pathway enrichment for Focal Adhesion Kinase FAK-PI3K-Akt–mTOR-signaling [14]. Gain in copy number of one of the lncRNAs in the ceRNA network (LINC00536) was found to correlate with poor prognosis. Five of the lncRNAs in the network were found to be related to immune infiltration and immune checkpoints. These effects on ceRNA regulation by genomic alterations have important implications on tumor progression and drug response. Angius et al. recently showed that miR-106b-25/miR-17-92 clusters are the most highly overexpressed miRNAs in basal-like breast cancer and that their expression is associated with CNV [29]. These findings indicate that genomic alterations are a substantial contributor to miRNA misexpression within breast cancer subtypes.

### 2.3. Gene Fusion and Translocation-Shaping ceRNAs and ceRNETs in TNBC

Fusion between genes that are both coding and non-coding can have substantial implications on the genomic profile in tumors, particularly that of characterizing tumor progression. Oncogenic fusion genes can result from chromosomal rearrangement, read-thorough errors or altered splicing [37]. While fusions in cancer can often result in an aberrant expression of oncogenic coding genes, they can also result in an aberrant generation or expression of non-coding genes with relevant regulatory functions. For example, a tumor-specific fusion, MRPS31-SUGT1, resulting from an intra-chromosomal translocation on chromosome 13, generates the previously identified lncRNA MRPS31P5 [38]. This fusion-generated lncRNA is implicated in a ceRNA network with several cancer-related miRNAs.

It has recently become evident that lncRNA genes are commonly subject to fusion with genes encoding other RNAs, including mRNAs and lncRNAs. In breast cancer, there has been documentation of several fusions involving the PVT1 lncRNA (plasmacytoma variant translocation 1), which resides in a fragile site on chromosome 8 and is implicated in a variety of cancers [39]. Amplification of PVT1 has been found to stabilize oncoproteins, including STAT3 and KLF5 [40,41]. STAT3 activation by PVT1 was shown to promote angiogenesis [41]. Specifically, in TNBC, PVT1 can promote tumorigenesis through the promotion of KLF5/β-catenin signaling [40].

Oncogenic circRNAs that promote transformation, survival and drug resistance can also be generated by chromosomal translocation/fusion (fusion circRNAs, f-circRNAs) [42]. F-circRNAs with oncogenic roles have been identified in other cancers. For example, the circRNA F-circEA1, which is generated by the EMF4/ALK1 fusion gene, acts as a miRNA sponge to promote tumorigenesis in non-small-cell lung cancer [43]. However, while F-circRNAs may very well play a role in breast cancer pathogenesis and progression, evidence for specific circular fusion transcripts that are specific to breast cancer is currently lacking.

### 2.4. Key Molecular Pathways Affected by Genomic Alterations in ceRNAs

The study of pathogenic expression that is regulated by aberrant ceRNA networks in cancer is a continuing endeavor that is growing as the significance and consequences of disruptions in this regulatory mechanism come to light. Much of the early study of ceRNA networks in cancer led to discoveries of modulation of PTEN levels in tumors that lead to cancer progression [44]. These discoveries have led to further discoveries of targets of ceRNA network regulation and the cancer-relevant molecular pathways that are affected. Recent studies have predicted that hundreds of cancer genes are aberrantly regulated in multiple cancer types, including breast cancer [45]. Here, we focus on the genomic alterations that result in pathogenic changes in the ceRNA networks in cancer, beginning with general mechanistic concepts and following specific alterations in TNBC in the next section.

**Figure 3:**
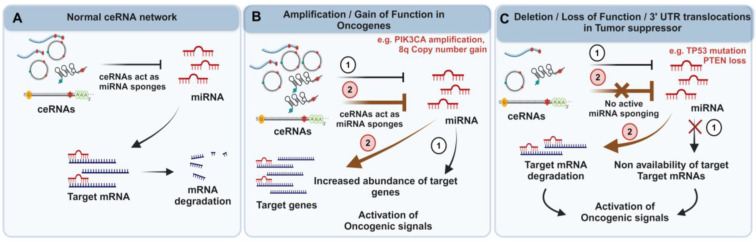
Interplay between genomic alterations and ceRNA networks: This figure illustrates how genomic alterations within ceRNAs or target genes may facilitate the activation of oncogenic signals. In normal conditions (**A**), ceRNAs function as miRNA sponges, effectively modulating miRNA control over target genes and thereby maintaining a delicate balance. However, in a cancerous state, due to elevated genomic alterations, the following two scenarios might result in the activation of oncogenic signals. The first scenario is amplification/gain of function (**B**), observed in the genomic locations of target oncogenes, e.g., PIK3CA amplification or 8q copy number gain will ultimately increase their abundance, activating their respective oncogenic signals (represented as (1)). On the other hand, amplification/gain of function alterations (**B**), observed in the genomic locations of ceRNAs, can effectively sponge miRNAs, resulting in the increased abundance of target oncogenes and the subsequent activation of oncogenic signals (represented as (2)). The second scenario is Deletion/Loss of Function/3′ UTR translocations in tumor suppressor regions (**C**), e.g., TP53 mutations or PTEN loss, which can signify the unavailability of these regions to target miRNAs. This unavailability activates oncogenic signals (represented as (1)). Similarly, deletion or loss of function alterations in ceRNAs eliminate their ability to actively sponge miRNAs. Consequently, miRNAs are free to bind to the target tumor suppressor genes, leading to their degradation and facilitating the effective activation of oncogenic signals (represented as (2)).

CNV and genomic deletions and amplifications can affect non-coding RNA expression as well as coding mRNA expression in cancers, affecting the ceRNA networks and, ultimately, oncogenic or tumor suppressor activity (Figure 3). Early in the study of ceRNA networks, several transcripts were identified as ceRNA competitors of miRNAs targeting PTEN, which can be downregulated in cancer, resulting in PTEN repression [44]. These include PTENP1, ZEB2 (Zinc Finger E-Box Binding Homeobox 2), CNOT6L, VAPA (VAMP-Associated Protein A) and PTENP1. In glioblastoma, copy number alterations in regions encoding PTEN-regulating non-coding ceRNAs were found, which they hypothesized may be responsible for decreased mature PTEN in tumors [45]. Further, a 3′UTR shortening of ceRNA transcripts affecting tumor suppressors, including PTEN, has been shown to disrupt the regulation of the ceRNETs that play a role in tumorigenesis [8].

As introduced earlier in this section, p53 regulates EMT in breast cancer through a ceRNA network, depending on its deletion or wild-type or mutant status [23]. EMT has also been found to be regulated in lung cancer cells by copy number-amplified miR-21, which targets FOXP1 using a single, highly conserved MRE [46]. Within a ceRNA network, miR-21 was shown to interact competitively with the TGFB1 transcript, which represented 90% of the increased miR-21 MRE during EMT in A549 cells. These examples of molecular pathways affected by the genomic alteration of ceRNAs in cancer represent a fraction of the mechanisms by which altered ceRNETs contribute to tumorigenesis and progression, including that in TNBC, although they may be well-characterized. Recent work is beginning to clarify the landscape of ceRNETs alteration and its molecular and pathologic effects in TNBC, bringing insights that are likely to impact the understanding and treatment of the disease. Among commonly altered ceRNA molecular targets and pathways, PTEN/pAkt, p53/DNA repair and EMT stand out as key cancer-related genomic alteration-affected pathways in TNBC, which, among other mechanisms, are discussed in more detail below.

## 3. Significance of ceRNETs Alterations in TNBC

**Figure 4:**
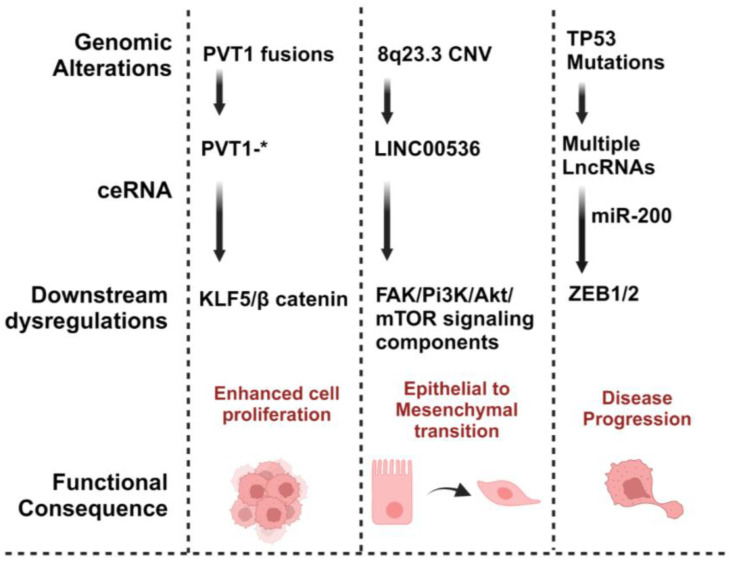
Genomic alterations of ceRNAs can affect clinical aspects of TNBC by driving the clinical complexities through their related ceRNA regulatory networks ceRNETs. Fusion events, for example, involving the lncRNA PVT1, regulate the downstream KLG5/β regulation, contributing to the enhanced cellular proliferation. (*) represents multiple genes involved in known fusions with PVT1. Copy number variations (CNVs) on 8q23.3 and its associated dysregulations among LINC00536 and lncRNA drive FAK/PI3k/Akt/mTOR signaling components, leading to the epithelial–mesenchymal transition (EMT) and escalate the metastatic potential. Additionally, TP53 mutation, coupled with multiple dysregulated lncRNAs, acting as miRNA sponges for miR-200, regulates the ZEB1/2 genes and consequently promotes TNBC progression.

Given the common defects in the DNA damage response (DDR) in TNBC, including alterations of BRCA1/2 and TP53, genomic instability and alterations play a large role in the progression of the disease [13]. Since the majority of the genome is non-coding, it is likely that a substantial fraction of genomic alteration in cancer resides in non-coding regions that have complex regulatory functions. Differences in the profiles of molecular functions and the clinical significance of ceRNAs between breast cancer subtypes are being explored. Indeed, ceRNA networks have been found to play important potential roles in TNBC development and progression [26]. As examples, many TNBC-specific circRNAs acting as ceRNAs affecting initiation and progression have been identified [4], as have ceRNETs involving lncRNAs, affecting the clinical outcome and prognosis of TNBC [47]. These act through pathogenic changes in EMT, invasion, migration and metastasis, among other mechanisms, with potential clinical implications and impact on biomarker discovery, target discovery and drug response [4,47]. Many of the specific ceRNAs that have been identified in TNBC and their clinical significance are discussed herein. Key examples of the genomic alterations of ceRNAs affecting TNBC phenotypes are illustrated in Figure 4.

### 3.1. Identification and Characterization of Key ceRNAs Molecules in TNBC

In breast cancer, regulatory mechanisms have been identified as involving multiple specific ceRNAs. The accumulation of mRNA of many cancer-related genes, including BCL2, CDKN1B, EGR1, FOS, N-RAS and RB1, were found to be altered in MCF7 cells in response to the silencing of predicted ceRNA regulators [45]. Approximately 450,000 ceRNA network interactions were identified in this study. Within these networks, predicted regulatory ceRNAs for cancer driver genes were tested, with 68% of the driver genes being downregulated upon silencing of their respective inferred ceRNA regulators. Zhu et al. showed that ceRNA networks are modulated by CNV in lncRNAs in breast cancer [14]. These networks were enriched in focal Adhesion FAK-PI3K-Akt–mTOR signaling. Several circRNAs have been identified as miRNA sponges in breast cancer [4]. These include circSEPT9, promoting LIF/Stat3 signaling [46]; hsa_circ_0000199, promoting PI3K/Akt/mTOR signaling [48]; circNR3C2, promoting HRD1 expression (HMG-CoA reductase degradation protein 1) and resulting in increased proliferation, EMT, migration and invasion [49]; and circ_0001667, promoting NCOA3 expression (nuclear receptor coactivator 3) and resistance to Adriamycin in breast cancer cells through competition for miR-4458 [4,50]. Another standout example of dysregulated ceRNA networks in breast cancer is the miR-200 family of miRNAs, which interacts with p53 in breast cancer and, as noted above, regulates the ceRNA networks controlling EMT and metastasis, partly through the regulation of ZEB1/2 [23]. With a large number of ceRNA network interactions regulating a variety of targets and pathways in breast cancer, it is valuable to delineate the subtype-specific profiles of ceRNA dysregulation and their effects on cancer phenotypes. Here, we discuss specific key examples of the ceRNAs identified in TNBC and their consequences on molecular and phenotypic dysregulation. These and other TNBC-relevant ceRNAs not discussed in detail in the text are listed in Table 2.

### 3.2. Key ceRNAs Driving Tumorigenesis and Disease Progression

Several ceRNAs have been implicated in TNBC tumorigenesis and malignant progression. The lncRNAs small nucleolar RNA host gene 12 (SNHG12), induced by c-Myc [85], highly upregulated in liver cancer (HULC), HOX transcript antisense intergenic RNA (HOTAIR) [83], and long intergenic non-protein-coding RNA-regulator of reprogramming (lincRNA-ROR) have altered expression in TNBC [2]. SNGH12 has been suggested as a therapeutic target for cancer [89]. These regulators are involved in tumorigenesis and progression via several mechanisms. For example, lincRNA-ROR interacts with miR-145 to increase EMT, invasion, metastasis and stemness in TNBC cells [88]. The regulation of EMT by ceRNAs is a recurring theme in TNBC, with lncRNAs NEAT1 (lncRNA nuclear enriched abundant transcript 1), HOTAIR and HULC also promoting a mesenchymal phenotype [2]. The latter is associated with metastasis and a poor outcome in TNBC [90] and has been shown to act through competition with miR-200a-3p in hepatocellular carcinoma [2,91], which is part of the miR-200 family/p53 axis of EMT regulation in breast cancer [23]. The lncRNA PVT1 promotes TNBC tumorigenesis through KLF5/β-catenin signaling [40]. As discussed above, several regulatory PVT1 fusions have been identified in breast cancer [39]. CircTADA2A, which is downregulated in TNBC tissues, promotes SOCS3 expression, metastasis and an aggressive oncogenic phenotype through competition with miR-203a [63]. Malignant progression of TNBC is inhibited by circFBXW7, which acts as a sponge of miR-197-3p and encodes a protein [64]. This circRNA suppresses proliferation and migration in TNBC cells through the upregulation of FBXW7 expression (F-Box and WD Repeat Domain Containing 7).

### 3.3. ceRNAs and ceRNETS in Drug Response and Resistance Mechanisms in TNBC

In addition to the pathogenic changes brought on by alterations in the ceRNA networks, the effects on response and resistance to anticancer drugs have also been observed. CircRNA/miRNA/mRNA interactions have been implicated in resistance to paclitaxel (PAX), such as CircGFRA1/miR-361-5p/TLR4, Circ-0006528/miR-1299/CDK8, and Circ-ABCB10/let7a-5p/DUSP7, and 5-fluorouracil, such as CDR1as/miR-7/CCNE1 and CircFBXL5/miR-216b/HMGA2, as well as doxorubicin and tamoxifen, such as CircBMPR2/miR-553/USP4, hsa_circ_0025202/mir-182-5p/FOXO3, and miR-197-3p/HIPK3, and in various cancers, including breast cancer and TNBC [92]. Moreover, the lncRNA FTH1P3 promotes paclitaxel resistance in TNBC cells through a miR-206/ABCB1 axis [2,93]. The BMP/OP-Responsive Gene (BORG), which is overexpressed in TNBC and associated with metastasis, also promotes doxorubicin cytotoxicity [91]. The stress-induced survival activity of BORG is dependent on NF-kB activation. HCP5, upregulated in TNBC, promotes cisplatin sensitivity through the regulation of PTEN expression and Akt phosphorylation (Table 2) [69]. LncRNA H19 is overexpressed in the majority of breast cancers, particularly in paclitaxel-resistant TNBC cells compared to sensitive cells (Table 2) [68]. Silencing of H19 led to apoptosis via decreased Akt phosphorylation [62]. These effects on drug response make ceRNAs potential targets for drug sensitization and potential markers of therapeutic responses. Given their involvement in drug response, investigation of their utility as markers of response is justified. Examples of evidence or the use of specific ncRNAs as markers of drug response, as well as those of prognosis, are discussed further below.

### 3.4. Clinical Implications of ceRNAs and ceRNETs in TNBC

Prevalent genomic alterations in TNBC affecting non-coding regions, particularly those that are translated into ceRNA species, can have important clinical implications on disease aggressiveness, survival outcomes and drug response. They may also provide novel biomarkers for the diagnosis, treatment and prognosis, and key dysregulated ceRNAs may be promising therapeutic targets. The field of ceRNA research is conceptually over a decade old, but in practice, it is just now burgeoning. Therefore, a better understanding of the central ceRNET players in TNBC that control these clinical aspects will be required in order to fully exploit them for clinical benefit.

### 3.5. Implications of ceRNA and ceRNET Alterations on Outcomes in TNBC

Various ceRNAs and ceRNETs have been implicated in disease progression and metastasis of TNBC, ultimately having an impact on survival outcomes. For instance, lncRNAs HOTAIR, CNV-altered LINC00536, ARNILA (AR negatively induced lncRNA), and circRNAs circPLK, circIFI30 and circLIF4A have been associated with poor prognosis in TNBC patients (Table 3) [14,55,73,82,93,94]. The relationship of ceRNAs to outcomes in TNBC is typically a result of the direct effects on disease progression, e.g., metastasis. As discussed previously, ceRNA networks with miR-200 family members as central factors in controlling EMT and metastatic phenotypes in TNBC and other cancers [24,46,95]. Specific lncRNAs have been found to affect or predict metastasis of TNBC to specific sites. For example, DLX6-AS1 (DLX6 antisense RNA 1) was found to promote distant bone metastasis in breast cancer through the modulation of miR-9-5p and miR-124-3p and the subsequent induction of Wnt/β-catenin signaling [96], HOTAIR has been found to promote lymph node metastasis [83], hsa_circ_102229 has been found to be associated with lung metastasis [62], and circKIF4a has been shown to promote brain metastasis through STAT3 signaling [56].

P53-mediated phenotypes have been shown to be regulated by ceRNA networks, which are affected by p53 mutation [23]. Importantly, p53 mutation has been shown to be associated with poor recurrence-free and overall survival in TNBC, depending on the type of mutation [22]. A specific example is the lncRNA HULC, a player in the miR-200/p53 network and DNA repair function, which is associated with poor prognosis in TNBC [90]. RAD51AP1 and TYMS, co-regulated with a ceRNA network in TNBC [26], have also been shown to be associated with poor overall survival and recurrence, respectively, in lung and prostate cancers, respectively [28,97]. More recently, RAD51AP1 was shown to be overexpressed and associated with poor relapse-free survival in TNBC, acting through the promotion of breast cancer stem cell renewal [98]. Several other ceRNAs have been implicated as influencing outcomes or as biomarkers in TNBC, as listed in Table 3.

**Table 3:** ceRNAs affecting clinical aspects of TNBC.

Type	ceRNAs	Clinical Implications	Reference
circRNA	**circ_0044234**	Prognosis	Darbeheshti et al., 2021 [99]
circRNA	**CircPLK1**	Poor Prognosis	Kong et al., 2019 [93]
circRNA	**circIFI30**	TNM Stage, Grade and Poor Prognosis	Xing et al., 2020 [94]
circRNA	**circKIF4A**	Poor Prognosis	Tang et al., 2019 [55]
circRNA	circGFRA1	Poor Survival	He et al., 2017 [100]
LncRNA	multiple	Poor Overall, Relapse-Free Survival, Recurrence	Liu., 2019 [46]
LncRNA	**HOTAIR**	Poor Survival	Liang., 2019 [82]
LncRNA	**LINC00536**	Poor Prognosis	Zhu., 2023 [14]
LncRNA	LINC01315	Prognostic Marker	Naorem., 2020 [20]
LncRNA	CTA-384D8.35	Prognostic Marker	Naorem., 2020 [20]
LncRNA	LINC01087	TNBC Diagnostic Marker over non-TNBC	Naorem., 2020 [20]
LncRNA	LINC01315	TNBC Diagnostic Marker over non-TNBC	Naorem., 2020 [20]
LncRNA	SOX9-AS1	TNBC Diagnostic Marker over non-TNBC	Naorem., 2020 [20]
LncRNA	MIR155HG	Marker of IO Therapy Response, Prognosis, Biomarkers to Predict Response to ICI Therapy	Peng., 2019 [101]
LncRNA	**DLX6-AS1**	Distant Bone Metastasis	Liu., 2020 [96]
LncRNA	**ARNILA**	Poor Prognosis	Yang et al., 2018 [73]

Note: ceRNAs in bold also have physiologic/pathologic functions in TNBC listed in Table 2.

### 3.6. Predictive Biomarkers for Outcomes in TNBC

In addition to directly affecting clinical outcomes, many ceRNAs may also be useful as markers of diagnosis, prognosis or treatment responses. Specific prognostic and theranostic ceRNA markers that have been identified are outlined in Table 3. As an example, miR-146a-5p was found to regulate a ceRNA network, including the lncRNA HOTAIR, and to be a predictive biomarker of poor prognosis in TNBC [89]. The effects of miR-146a-5p on the migration and invasion of TNBC cells were dependent on the upregulation of HOTAIR. The construction of regulatory networks in TNBC has revealed distinct ceRNA networks with specific targets that predict prognosis [10,102]. In a study of differentially expressed lncRNAs in TNBC, LINC01315 and CTA-384D8.35 were identified as potential prognostic markers, and LINC01087, LINC01315 and SOX9-AS1 were found to differentiate TNBC from non-TNBC [20]. Among mRNAs competitively regulated by DLX6-AS1, a lncRNA introduced above, FBN3, JGB3, PTPRZ1, FBN3 and CAMGV were regulated through various miRNAs and were found to be associated with poor prognosis in TNBC [96]. These appear to be part of the larger network implicating a DLX6-AS1/WNT6 axis in distant bone metastasis [96]. In addition, CNV-altered LINC00536 was shown to be associated with poor prognosis in breast cancer patients [14].

### 3.7. Regulation of Drug Response by ceRNAs in TNBC

**Figure 5:**
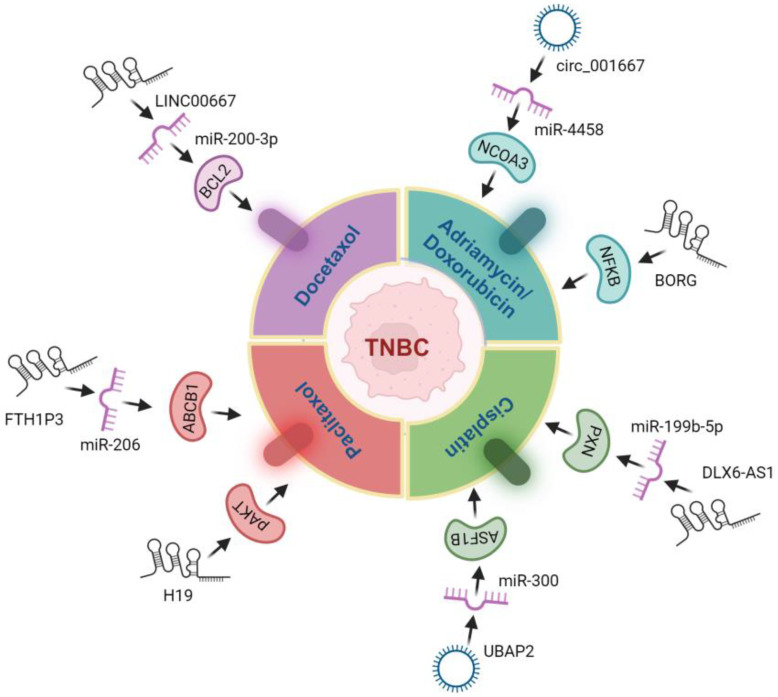
ceRNAs/ceRNETs and associated chemoresistance in triple-negative breast cancer (TNBC): Several ceRNAs have been reported as influential factors in mediating chemoresistance in TNBC. This figure gives an overview of the ceRNA-mediated resistance mechanisms encountered by various chemotherapeutic agents, such as taxanes (Paclitaxol, Docetaxol), and anthracyclines, such as doxorubicin and platinum-based drugs like (cisplatin) in TNBC.

Xia et al. recently highlighted the importance of non-coding RNAs as mechanisms of chemoresistance in TNBC [103]. They reviewed many miRNAs, circRNAs and lncRNAs that have implications on chemoresistance in TNBC and hypothesized that ncRNA profiling will be a viable means of selecting a chemotherapy that is likely to garner a response. Examples in the works of literature of ceRNAs that affect the response to chemotherapies include circ_0001667, conferring Adriamycin resistance; LINC00667, conferring docetaxel resistance; BORG, conferring doxorubicin resistance; UBAP, HCP5 and DLX6-AS1, conferring cisplatin resistance; and FTH1P3 and H19, conferring paclitaxel resistance (Table 2) [50,51,66,67,68,69,70,71]. Given the complicated mechanisms of chemoresistance with many effector proteins playing a role, targeting key ceRNA regulatory networks to cover multiple mechanisms or as more reliable markers that are based on a greater number of functional factors may be promising. Chemotherapeutic response relationships to ceRNAs in TNBC are highlighted in Figure 5. Aside from the implications on conventional chemotherapy, ceRNA regulation can affect the tumor immune microenvironment (TIME) of TNBC tumors and the effectiveness of immunotherapy. Five breast cancer CNV-altered lncRNAs were recently found to be related to immune infiltration and immune checkpoints with implications on the selection and use of the immune checkpoint blockade [14]. The lncRNA MIR155HG was also found to be associated with immune infiltration and prognosis after immunotherapy in multiple cancer types [101], demonstrating that these regulators may be fundamental players in and indicators of the response to immunotherapy and tumor immune status.

## 4. Conclusions and Future Directions

The study of ceRNA networks can be exceedingly complex, especially given that ceRNA species are thought to have functions in regulating transcription and translation by multiple proposed mechanisms in addition to the competitive inhibition of miRNAs [15]. While this work may be challenging, much has been learned about the ceRNAs and ceRNETs that have pathogenic roles and/or therapeutic implications in TNBC. It is also clear that the regulatory profiles of TNBC are somewhat unique among cancers and breast cancer subtypes, being enriched in functions and pathways that are central to TNBC pathogenesis. The progress presented herein, detailing the specific TNBC-associated ceRNA network changes resulting from genomic alterations, which are common to the disease, provides a roadmap to future exploitation of these factors for an improved mechanistic understanding, therapy and outcomes for TNBC patients.

In 2016, Wang et al. presented the outstanding questions regarding ceRNA networks in cancer [104]. Substantial progress has been made on some fronts, specifically regarding how ceRNA crosstalk can be exploited by cancer cells, how they contribute to therapeutic resistance mechanisms and how they change during tumor evolution. However, there is much to be learned, and clinical implementation of the knowledge we have gained regarding ceRNAs in cancer has yet to be accomplished. Another question that remains is: What are the baseline and disease-associated differences in ceRNA crosstalk that are attributable to genetic heterogeneity? Large-scale studies may uncover the answer.

Disease-relevant genomic alterations may be occurring in regions of the genome that do not contain known pro- or anti-tumorigenic factors but, rather, contain regulatory ceRNAs that affect the expression of multiple effector genes. These mutations, splice variants and copy number variations (CNV) may have been previously dismissed as inconsequential, although they may conceal genes that code for truly disease-altering regulatory RNAs. Re-analysis of existing sequencing data and new studies covering regions of the genome that may lack a protein-coding sequence will be required to finally and thoroughly identify altered ceRNAs in cancer and their consequences on cancer pathogenesis and outcomes, leading to significant clinical implications.

## Figures and Tables

**Table 1:** Most frequently mutated genes in triple-negative breast cancer (TNBC) subtypes, with genes having more than 10% mutation frequency.

	Mutation Frequency							
Gene	Cytoband	TNBC	BL1	BL2	IM	LAR	M	MSL
TP53	17p13.1	78.20%	76.50%	88.20%	80.60%	76.50%	80.00%	68.20%
PIK3CA	3q26.32	11.70%	5.90%	17.60%	13.90%	47.10%	-	4.50%
PTEN	10q23.31	6.70%	2.90%	17.60%	11.10%	17.60%	-	4.50%
KMT2D	12q13.12	6.10%	5.90%	5.90%	2.80%	17.60%	7.50%	4.50%
KMT2C	7q36.1	5.60%	2.90%	5.90%	2.80%	11.80%	7.50%	-
TPR	1q31.1	5.00%	11.80%	-	2.80%	11.80%	2.50%	-
BRCA1	17q21.31	5.00%	2.90%	11.80%	5.60%	-	5.00%	4.50%
ARID1B	6q25.3	5.00%	2.90%	17.60%	5.60%	5.90%	2.50%	4.50%
CREBBP	16p13.3	5.00%	11.80%	-	2.80%	-	7.50%	4.50%
RNF213	17q25.3	4.50%	5.90%	-	5.60%	11.80%	2.50%	-
MED12	Xq13.1	4.50%	8.80%	-	2.80%	17.60%	-	-
PCLO	7q21.11	3.90%	8.80%	11.80%	-	5.90%	2.50%	-
NOTCH4	6p21.32	3.40%	2.90%	11.80%	2.80%	5.90%	2.50%	-
MGA	15q15.1	2.80%	2.90%	-	2.80%	11.80%	2.50%	-
UBR5	8q22.3	2.80%	5.90%	-	-	11.80%	2.50%	-
ERBB2	17q12	2.80%	-	-	2.80%	23.50%	-	-
CPS1	2q34	2.80%	-	5.90%	2.80%	11.80%	-	-
PDE4DIP	1q21.2	2.80%	5.90%	5.90%	-	11.80%		
ERBB4	2q34	2.80%	11.80%	5.90%	-	-	-	-
SLIT2	4p15.31	2.20%	2.90%	-	-	-	-	13.60%
ASXL2	2p23.3	2.20%	-	-	-	-	10.00%	-
EGFR	7p11.2	2.20%	-	-	2.80%	11.80%	-	4.50%
PER1	17p13.1	2.20%	2.90%	-	-	11.80%	-	-
CUL4A	13q34	1.70%	-	-	-	11.80%		4.50%
EIF4A2	3q27.3	1.70%	-	-	-	11.80%	2.50%	-
ARID4A	14q23.1	1.70%	-	-	-	11.80%	2.50%	-
CDH1	16q22.1	1.70%	-	-	2.80%	11.80%	-	-
AMER1	Xq11.2	1.70%	-	-	2.80%	11.80%	-	-
PALB2	16p12.2	1.70%	-	-	2.80%	11.80%	-	-
ZMYM3	Xq13.1	1.70%	-	-	-	11.80%	-	-
ARID4B	1q42.3	1.70%	2.90%	11.80%	-	-	-	-
NBN	8q21.3	1.70%	2.90%	11.80%	-	-	-	-
INSR	19p13.2	1.10%	-	11.80%	-	-	-	-
JAK3	19p13.11	1.10%	-	11.80%	-	-	-	-

**Table 2:** Physiologic and pathologic functions of key ceRNAs in TNBC.

Type	ceRNA	miRNAs	Target Transcripts	Physiologic/Pathologic Functions	Reference
circRNA	circSEPT9	miR-637	LIF	LIF/Stat3 Signaling, Migration, Invasion, Proliferation	Zheng 2020 [51], Wang et al., 2023 [4]
circRNA	circ_0001667	miR-4458	NCOA3	Adriamycin Resistance	Cui et al., 2022 [50]
circRNA	circINTS4	miR-129-5p	POM121	ADR Resistance	Tang et al., 2022 [52]
circRNA	circUBAP2	miR-300	ASF1B	Cisplatin Resistance, Regulates PI3K/AKT	Wang et al., 2022 [53]
circRNA	circEPSTI1	miR-4753, miR-6809/	BCL11A	Proliferation, Apoptosis	Chen et al., 2018 [54]
circRNA	circKIF4A	miR-375	KIF4A	Proliferation and Migration	Tang et al., 2019 [55]
circRNA	circKIF4A	miR-637	STAT3	Brain Metastasis	Wu et al., 2024 [56]
circRNA	ciRS-7	miR-1299	MMPs	Migration and Invasion	Sang et al., 2018 [57]
circRNA	circZEB1	miR-448	eEF2K	Proliferation	Pei et al., 2020 [58]
circRNA	circNR3C2	miR-513a-3p	HRD1	Proliferation, Migration, Invasion, EMT	Fan 2021 [49]
circRNA	circAHNAK1	miR-421	RASA1	Inhibits Proliferation and Metastasis	Xiao et al., 2019 [59]
circRNA	circRAD54L2	miR-888	PDK1	Invasion, Metastasis, Proliferation	He et al., 2023 [60]
circRNA	circWAC	miR-142	WWP1	Paclitaxel Resistance, PI3K/Akt Pathway, Poor Prognosis, Proliferation and Invasiveness	Wang et al., 2023 [4]
circRNA	circ_0000199	miR-613, miR-206	PI3K/Akt/mTOR	Proliferation, Migration, Invasion and Chemo-Sensitivity	Li et al., 2021 [48]
circRNA	hsa_circ_0006220	miR-197-5p	CDH19	Tumor Suppressor Role	Shi et al., 2021 [61]
circRNA	hsa_circ_102229	miR-152-3p	PFTK1	Tumorigenesis, Lung Metastasis	Du et al., 2021 [62]
circRNA	circTADA2A-E6	miR-203a-3p	SOCS3	Proliferation, Migration, Invasion	Xu et al., 2019 [63]
circRNA	circFBXW7	miR-197-3p	FBXW7	Inhibits Migration and Proliferation	Ye et al., 2019 [64]
LncRNA	CCAT1	miR-17-5p	PDL1	Atezolizumab Resistance	Selem et al., 2023 [65]
LncRNA	FTH1P3	miR-206	ABCB1	Paclitaxel Resistance	Wang et al., 2018 [66]
LncRNA	BORG	-	NF-kB signaling, RPA1	Doxorubicin Resistance, Metastasis	Gooding et al., 2019 [67]
LncRNA	H19	-	pAkt-AKT signaling pathway	Paclitaxel Resistance	Han et al., 2018 [68]
LncRNA	HCP5	-	PTEN	Cisplatin sensitivity via PTEN/pAkt	Wu et al., 2019 [69]
LncRNA	DLX6-AS1	miR-199b-5p	PXN	EMT, Cisplatin resistance	Du et al., 2020 [70]
LncRNA	LINC00667	miR-200-3p	BCL2	Docetaxel Resistance	Li et al., 2022 [71]
LncRNA	HULC	miR-200a-3p	p53	p53, DNA Repair, Mesenchymal Phenotype	Das et al. 2023 [2]
LncRNA	SOX2-OT	miR-942-5p	PIK3CA	Activates PI3k/Akt, Activates Metastasis	Zhang et al., 2022 [72]
LncRNA	ARNILA	miR-204	SOX4	EMT, Invasion, Metastasis	Yang et al., 2018 [73]
LncRNA	HCP5	miR-219a-5p	BIRC3	Proliferation	Wang et al., 2019 [74]
LncRNA	DUXAP8	miR-29a-3p	SAPCD2	Promotes Proliferation, Suppresses Apoptosis	Yang et al., 2021 [75]
LncRNA	ST8S1A6-AS1	miR-145-5p	Activates CDCA3 and Inactivates p53/p21 signaling	Proliferation, Metastasis and Invasion	Qiao et al., 2022 [76]
LncRNA	LRRC75A-AS1	miR-380-3p	BAALC	Proliferation, Invasion, EMT	Li et al., 2020 [77]
LncRNA	SNHG6	miR-125b-5p	BMPR1B	Proliferation, Migration, Apoptosis	Lv et al., 2021 [78]
LncRNA	LRP11-AS1	miR-149-3p	NRP2	Tumorigenesis, Metastasis	Li et al., 2022 [79]
LncRNA	LincRNA-RoR	miR-145	MUC1	Invasion, Metastasis	Ma et al., 2018 [80]
LncRNA	DANCR	miR-874-3p	SOX2	Invasion	Wu et al., 2020 [81]
LncRNA	HOTAIR	miR-146a-5p	-	Lymph Node Metastasis, LAR Subtype	Liang 2019 [82], Collina 2019 [83]
LncRNA	HOST2	let-7b	STAT3	Proliferation, Migration	Hua et al., 2020 [84]
LncRNA	SNHG12	-	MMP13	Proliferation, Migration, Apoptosis	Wang et al., 2017 [85]
LncRNA	SENP3	miR-195-5p	EIF4A1/CCNE1	Progression	Chen et al., 2021 [86]
LncRNA	HNF1A-AS1	miR-32-5p	RNF38	Progression	Yang et al., 2021 [87]
LncRNA	PVT1	-	KLF5/β-catenin	Tumorigenesis	Tang et al., 2018 [40]
LncRNA	LincRNA-ROR	mir-205	ZEB2	EMT, Invasion, Metastasis, Stemness	Hou et al., 2014 [88]
miRNA	miR-200 family	miR-200 family	p53, EMT-TFs such as ZEB1/2	EMT, Metastasis	Parfenyev et al., 2021 [23]

## Data Availability

Not applicable.
